# Performance and microbial community analysis on nitrate removal in a bioelectrochemical reactor

**DOI:** 10.1371/journal.pone.0290660

**Published:** 2023-09-14

**Authors:** Han Li, Ying Cui, Fei Wang, Jinghua Li, Dafu Wu, Jing Fan

**Affiliations:** 1 School of Environment, Henan Normal University, Xinxiang, Henan, P. R. China; 2 School of Resource and Environmental Sciences, Henan Institute of Science and Technology, Xinxiang, Henan, P.R. China; 3 Key Laboratory of Yellow River and Huai River Water Environment and Pollution Control, Ministry of Education, Xinxiang, Henan, P. R. China; 4 Henan Key Laboratory for Environmental Pollution Control, Xinxiang, Henan, P. R. China; Universidade de Coimbra, PORTUGAL

## Abstract

In this experiment, we took reflux sludge, sludge from an aeration tank, and soil from roots as microbial inoculating sources for an electrochemical device for denitrification with high-throughput sequencing on cathodic biofilms. The efficiency of nitrate nitrogen removal using different microbial inoculates varied among voltages. The optimal voltages for denitrification of reflux sludge, aeration tank sludge, and root soil were 0.7V, 0.5V, and 0.5V, respectively. Further analysis revealed that the respective voltages had a significant effect upon microbial growth from the respective inoculates. *Proteobacteria* and *Firmicutes* were the main denitrifying microbes. With the addition of low current (produced by the applied voltage), the Chao1, Shannon and Simpson indexes of the diversity of microorganisms in soil inoculation sources increased, indicating that low current can increase the diversity and richness of the microorganisms, while the reflux sludge and aeration tank sludge showed different changes. Low-current stimulation decreased microbial diversity to a certain extent. *Pseudomonas* showed a trend of decline with increasing applied voltage, in which the MEC (microbial electrolysis cell) of rhizosphere soil as inoculates decreased most significantly from 77.05% to 12.58%, while the MEC of *Fusibacter* showed a significant increase, and the sludge of reflux sludge, aeration tank and rhizosphere soil increased by 31.12%, 18.7% and 34.6%, respectively. The applied voltage also significantly increased the abundance of *Azoarcus* in communities from the respective inoculates.

## 1. Introduction

With the rapid global economic development and growth of human activities, nitrogen pollution has gradually become a global environmental problem [1]. Excessive concentrations of nitrate-nitrogen have severely impacted not only human health, but also bodies of water, with detrimental effects including the eutrophication of rivers and lakes, the reduction of microbial diversity, and increased frequencies of cancers in animal organs and tissues [2]. In recent years, there has been a marked increase in the total volume of wastewater discharge in China [3–5]. Consequently, there has been a considerable shift of attention toward developing effective and environmentally sound methods of treating nitrogen-based constituents in sewage [6–9].

The traditional processes that are widely used to denitrify wastewater include physical processes, such as ion exchange; chemical processes, such as the conversion of nitrate nitrogen into ammonia via chemical reactions; and biological processes, involving a two-step process of nitrification and denitrification [10]. Although these approaches reliably remove a considerable amount of the nitrogen, these methods share the disadvantages of high operating cost, high energy consumption, and the generation of secondary pollution caused by intermediate products [11, 12]. The BES (Bio-Electrochemical System) was created to improve and optimize the traditional biotechnologies, and has been applied widely in contaminant removal due to its high treatment efficiency, low energy consumption, simplicity of operation, and low surplus sludge yield [13–17]. A BES is comprised of microbial fuel cells (MFCs) and microbial electrolysis cells (MECs). The main mechanism in MECs is the release of electrons to the anodes by the anode-oxidized matrix of electrochemically-active microorganisms. Subsequently, electrons reach the cathode via the circuit with the applied voltage to initiate the reduction reaction in the cathode chamber with the electron acceptor. Furthermore, by virtue of the microorganisms that they contain, MECs have stimulating mechanisms that accelerate contaminant removal [18]. In recent years, MECs have been applied widely in processes such as denitrifying artificial wetlands, hydrogen production in waste refineries, and seawater desalination, offering the dual benefits of decontamination and increased production capacity, particularly in the denitrification treatment of sewage [19]. Nguyen et al. [20] have employed a dual-chamber MEC with graphite felt as the electrode to treat synthetic nitrate wastewater, attaining a 79% nitrate removal rate. However, studies focusing on the effects of microbial inoculation on nitrate removal and microbial communities under different voltages in a bioelectrochemical reactor are few [21, 22]. High-throughput DNA sequencing, which is known widely as the next-generation sequencing technology, has been used extensively in the field of molecular biological detection. High-throughput sequencing can determine hundreds of thousands to millions of DNA sequences at a time, with fast sequencing speeds and high accuracy. Currently, high-throughput sequencing has been used extensively in determining the flora involved in denitrification [23].

In this context, we used a dual-chamber MEC to carry out a series of denitrification-based removals of nitrogen from simulated wastewater samples, using the soil from peony root systems, reflux sludge, and sludge from an aeration tank as our inoculation sources. For electrode materials and carbon source, we selected carbon blocks and C_4_H_4_Na_2_O_4,_ respectively. To understand the mechanisms effecting nitrate-nitrogen removal efficiency using an MEC at different voltages, we employed high-throughput DNA sequencing to analyze the microbial diversity on the carbon felt in the cathode chamber.

## 2. Materials and methods

### 2.1. The main reagents and microbial inoculating sources

The main reagents used in the experiment were PBS (phosphate buffered saline) [24] and a mineral solution. Minerals consisted of EDTA (50 mg/L), MnSO_4_·H_2_O (4.32 mg/L), FeSO_4_·7H_2_O (5.00 mg/L), CaCl_2_·2H_2_O (4.15 mg/L), CoCl_2_·6H_2_O (1.61 mg/ L), ZnSO_4_ (2.20 mg/L), CuSO_4_·5H_2_O (1.57 mg/L), H_3_BO_3_ (0.1 mg/L), and Na_2_MoO_4_ (1.1 mg/L).

This study employed three sources of microbial inoculation: reflux sludge, sludge from an aeration tank, and soil from roots. We obtained the reflux sludge and aeration tank sludge from a wastewater treatment plant in Xinxiang. The aeration tank sludge had not been acclimatized, making it immediately usable for inoculating experiments. The rhizosphere soil was taken from *peonia lactiflora* roots at the Experimental Base of Peony in Henan University of Science and Technology, Luoyang, Henan Province, China (34°33′ N, 112°16′ E). Sampling was conducted according to the methods described by Han et al. [25]. with some modifications. We removed the plant residues on the surface of the soil, and used a soil drill to collect soil from 5 sample points at 10 cm around the main stem of each peony by taking a core (5 cm in diameter and 20 cm in depth) from each sample point. This soil required treatment before being ground into a fine powder and stored in a refrigerator at 4°C. Before starting the experiment, the soil was made into a solution, placed into a shaker, and shaken for 30 minutes. We used the resulting solution as the microbial inoculating source from root soil.

### 2.2. Experimental device and start-up

**Fig 1:**
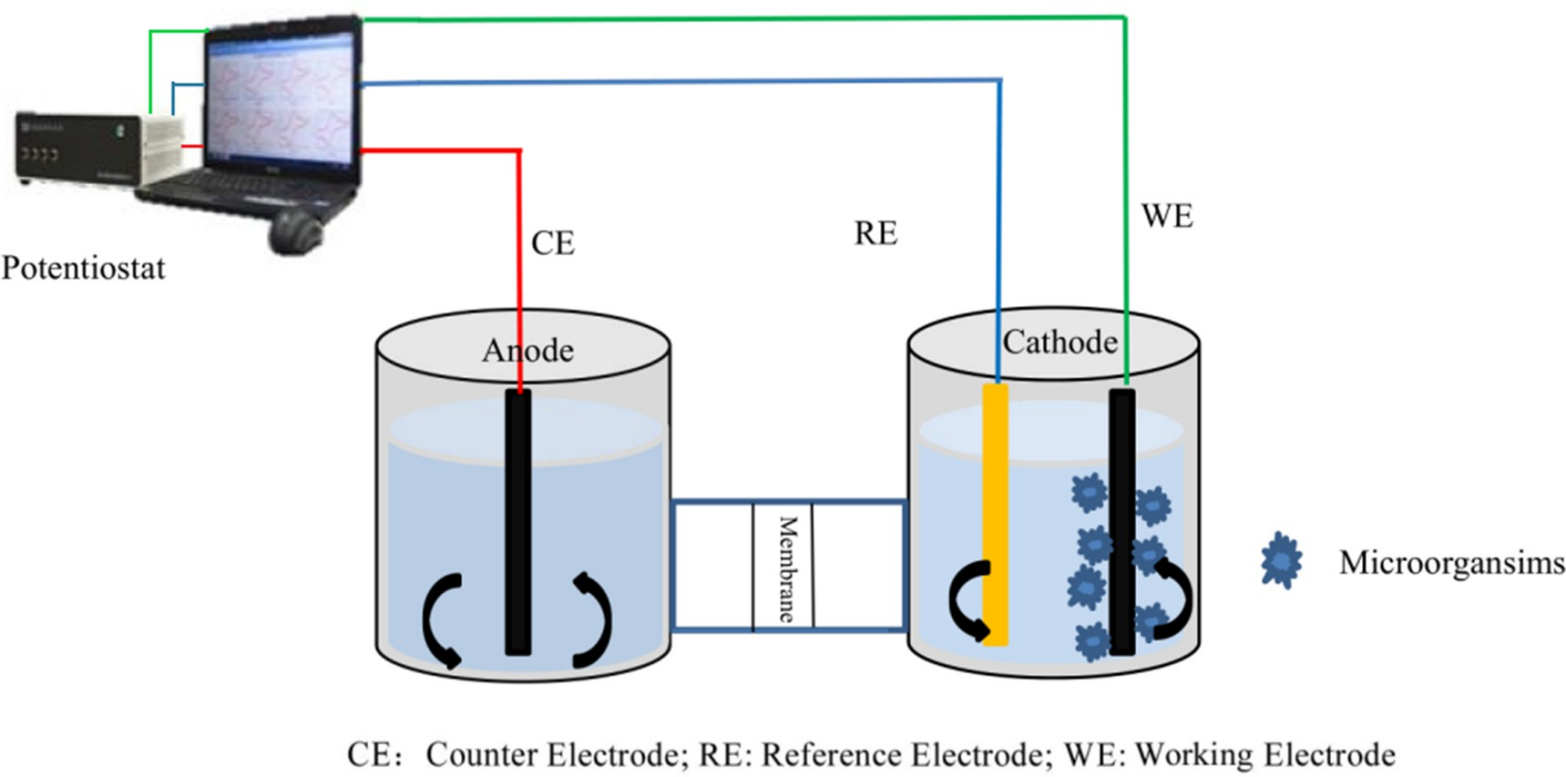
Dual-chamber MEC reactor. CE: Counter Electrode; RE: Reference Electrode; WE: Working Electrode.

As shown in Fig 1, the main reactor was a dual-chamber MEC reactor. Initially, the carbon felt was placed into boiling deionized water for two hours, after which it was air-dried, coiled with conductive wires, and placed in the cathode chamber of the reactor. It should be noted that the carbon felt was placed into the cathode chamber to increase the attachment of the microorganisms. The cathode chamber housed a magnetic rotor-powered stirrer, upon which the tank containing the simulated wastewater sample was placed and stirred to ensure uniform contact between the simulated wastewater and the microbial membranes. We then placed a carbon block in the anode chamber. The two reaction chambers were then integrated as a whole through the treated PEM (Proton Exchange Membrane, Nafion®N-117 membrane, 0.180mm thick, >0.90 meq/g exchange capacity, CAS:31175-20-9, Alfa Aesar chemical Co., Ltd). The whole device was cable-connected to a potentiostat that was, in turn, connected to a computer for controlling the voltage and detecting current changes.

The simulated wastewater sample used in this experiment contained Na_2_HPO_4_ (1.8 g/L), NaH_2_PO_4_ (0.66 g/L), KCl (0.1 g/L), C_4_H_4_Na_2_O_4_ (8 g). /L), KNO_3_ (1 g/L), and mineral solution (1.1 mL/L). We first added 30mL of the reflux sludge and 70mL of the simulated wastewater mixture into the cathode chamber, after which we added 100mL PBS into the anode chamber. After placing the device on the adjustable magnetic stirrer with heating function, the cathode and anode were connected. We then turned on the potentiostat and set the sampling time to 24 hours. The four voltages were set as 0.3 V, 0.5 V, 0.7 V, and 0.9 V, respectively. The voltage devices utilized were operated for three days under the same conditions, and the wastewater were sampled and stored at 4°C on a daily basis. After three days’ operation, a small piece of the carbon felt was cut and stored at -20°C to assess the types of microorganisms on it. The procedures that we employed for the aeration tank sludge and root soil inoculating sources were very similar to those used for the reflux sludge. All reactions were conducted at 30°C for 3d. Table 1 describes the operating conditions of the device.

**Table 1:** Operating conditions of MEC reactor.

Microbial inoculates	Reaction No.	Operating voltage
Reflux sludge	R2VI	0.3 V
	R2V2	0.5 V
	R2V3	0.7 V
	R2V4	0.9 V
Sludge from the aeration tank	A2VI	0.3 V
	A2V2	0.5 V
	A2V3	0.7 V
	A2V4	0.9 V
Soil from the roots	S2VI	0.3 V
	S2V2	0.5 V
	S2V3	0.7 V
	S2V4	0.9 V

### 2.3. DNA extraction, PCR amplification, and Illumina MiSeq sequencing

For sampling, biomass was collected from the MEC reactor at the end of each reaction. The collection procedure was repeated three times for each specimen. Genomic DNA from each sample (2.0 mL) was extracted using the FastDNA® SPIN Kit for Soil (Mp Biomedicals, Illkirch, France) according to the manufacturer’s protocols. The triplicate DNA samples extracted for each phase were pooled to generate one homogenized single genomic DNA sample. Prior to sequencing, the purified DNA was amplified by PCR with 338F (5’-ACTCCTACGGGAGGCAGCA-3’) and 806R (5’-GGACTACHVGGGTWTCTAAT-3’) primers for the hyper-variable V3-V4 region of the bacteria [26]. PCR amplification was performed in 25μL reaction mixtures containing 12.5 μL Phusion hot-start flex 2× master mix, 2.5 μL of each primer, 50ng template DNA, and ddH_2_O to adjust the volume. The cycling conditions for the PCR comprised a pre-denaturation step at 98°C for 30s, 35 cycles of 98°C for 10s, 54°C for 30s, and 72°C for 45s, then a final extension at 72°C for 10 min. PCR amplicons were purified with Vazyme VAHTSTM DNA Clean Beads (Vazyme, Nanjing, China) and quantified using the Quant-iT PicoGreen dsDNA Assay Kit (Invitrogen, Carlsbad, CA, USA). After the individual quantification step, amplicons were pooled in equal amounts, and pair-end 2× 250 bp sequencing was performed using the Illlumina MiSeq platform with MiSeq Reagent Kit v3 at Shanghai Personal Biotechnology Co., Ltd (Shanghai, China) [27].

### 2.4. Analysis methods

The samples were taken from the reactor on a daily basis. Samples were filtered through a 0.22-μm pore diameter membrane and were analyzed immediately. The nitrate-nitrogen content in the daily samples was measured using spectrophotometry, and the nitrite-nitrogen content was measured via spectrophotometry, with ethylene diamine dihydrochloride as the coupling agent [28].

Microbiome bioinformatics were performed with QIIME2 2019.4 [29] with slight modification according to the official tutorials (https://docs.qiime2.org//2019.4/tutorials/). Briefly, raw sequence data were demultiplexed using the *demux* plugin following by primers-cutting with *cutadapt* plugin [30]. Sequences were then merged, quality filtered and dereplicated using functions of fastq_mergepairs, fastq_filter and derep_fullength in the *Vsearch* plugin. All the unique sequences were then clustered at 98% (via cluster_size) followed by chimera removing (via uchime_denovo). At last, the non_chimera sequences were re_clustered at 97% to generate OTU representive sequences and the OTU table. Sequence data analyses were mainly performed using the QIIME2 and R packages (v3.2.0) [31]. OTU-level alpha diversity indices, such as the Chao1 richness estimator, Shannon diversity index, and Simpson index were calculated using the OTU table in QIIME2 and visualized as box plots. OTU-level ranked abundance curves were generated to compare the richness and evenness of OTUs among samples. Beta diversity analysis was performed to investigate the structural variation of microbial communities across samples and visualized via principal coordinate analysis (PCoA) [29, 32–34].

All statistical analyses were performed with IBM SPSS Statistics 26 for Windows (IBM Corp., Armonk, NY, USA). A *P*-value < 0.05 was considered to be statistically significant. Two-factor analysis of variance combined with one-way analysis of variance and *T* test were used for comparison.

## 3. Results and discussion

### 3.1. Determination of the optimum voltage

**Fig 2:**
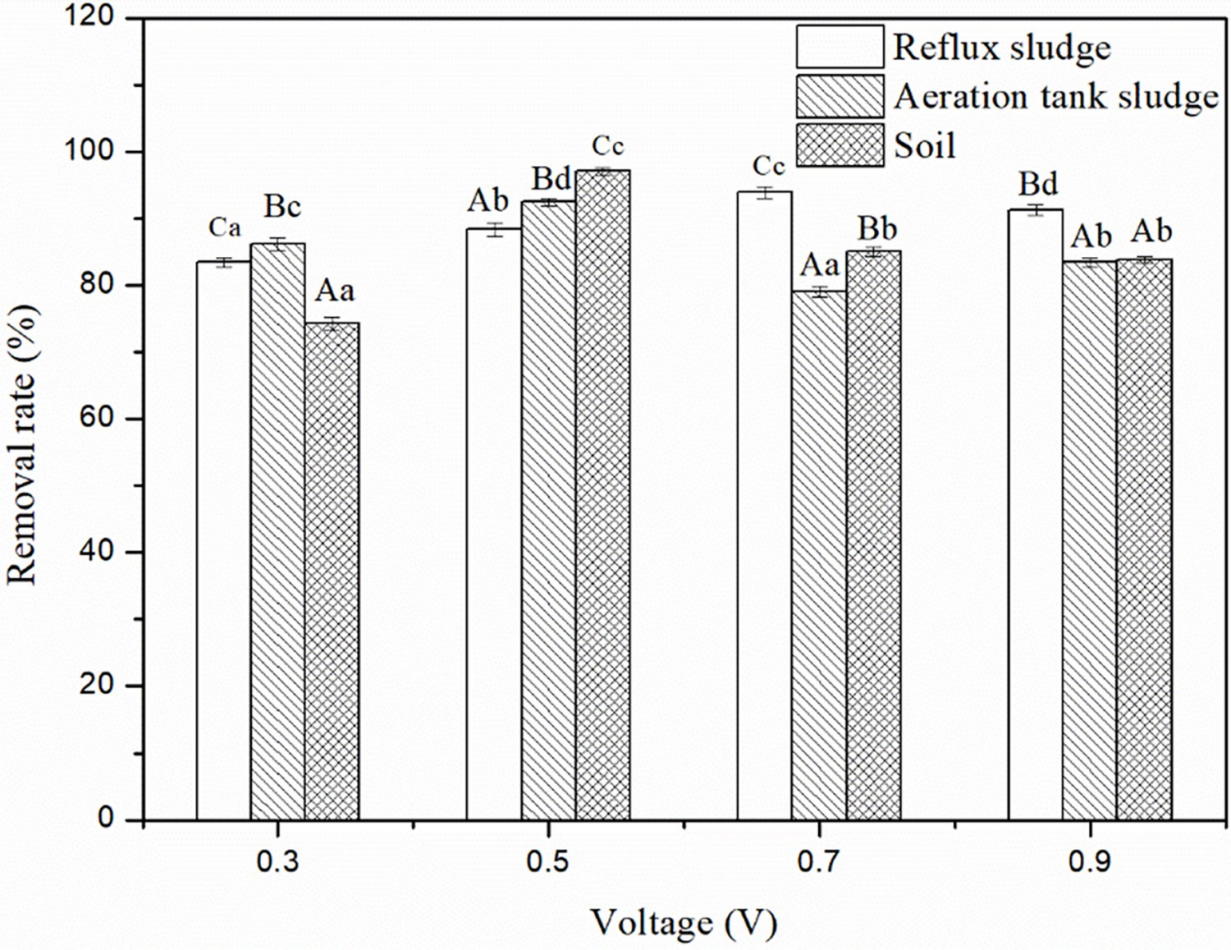
Nitrate-nitrogen removal efficiencies for different microbial inoculates at different voltages. Data in the figure are represented as the mean (n = 3) ± standard error (SE). The different uppercase letters on each bar indicate significant differences (*P* < 0.05) between different inoculation sources for the same voltage, and different lowercase letters on each bar indicate significant differences (*P* < 0.05) between different voltages for the same inoculate.

We conducted the nitrate-nitrogen removal experiment with the MEC device using reflux sludge, sludge from the aeration tank, or soil from peony roots as microbial inoculates at four different voltages (0.3 V, 0.5 V, 0.7 V, or 0.9 V), and determined the nitrogen removal efficiency. Fig 2 shows that the removal rate for nitrate-nitrogen varied among microbial inoculates of the same voltage. When we performed MEC denitrification with reflux sludge as the microbial inoculating source, the nitrate-nitrogen removal rates were 83.4%, 88.4%, 93.9%, and 91.3%, respectively, and the highest nitrate-nitrogen removal rate occurred at 0.7 V. When we performed the MEC denitrification on the aeration tank sludge, the nitrate nitrogen removal rates were 86.2%, 92.4%, 79.1%, and 83.4%, respectively, with the highest nitrate-nitrogen removal rate occurring at 0.5 V. For MEC denitrification using root soil as the inoculate, the nitrate-nitrogen removal rates were 74.3%, 97.1%, 85.0%, and 83.8%, respectively, with the highest nitrate nitrogen-removal rate occurring at 0.5 V.

**Fig 3:**
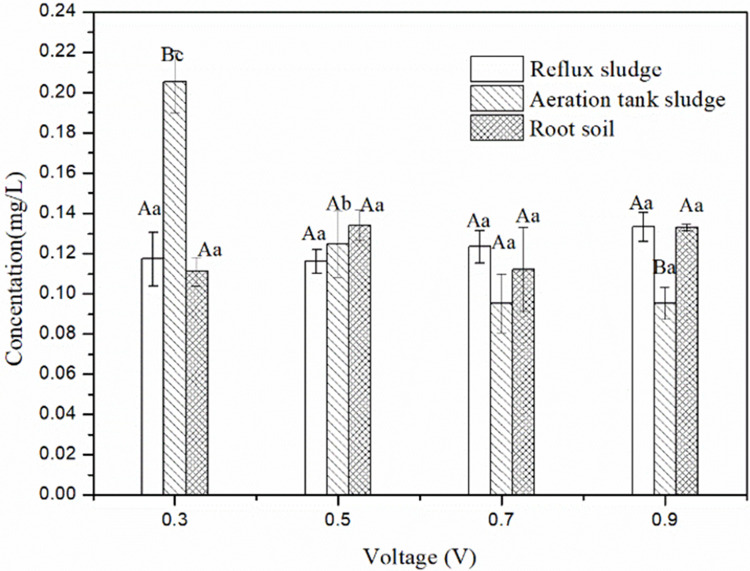
Nitrite-nitrogen concentrations with different microbial inoculates at different voltages. Data in the figure are represented as the mean (n = 3) ± standard error (SE). The different uppercase letters on each bar indicate significant differences (*P* < 0.05) between different inoculation sources for the same voltage, and different lowercase letters on each bar indicate significant differences (*P* < 0.05) between different voltages for the same inoculate.

During denitrification, nitrate-nitrogen is first converted to nitrite-nitrogen and then converted to nitrogen. Hence, nitrite-nitrogen concentration in the denitrification reaction is an indicator for determining optimum denitrification voltage. Fig 3 shows that the concentration of nitrite-nitrogen involving different voltages was 1.2±0.1 mg/L on average, and the optimum voltage was 0.7 V when we used reflux sludge as the inoculate. When aeration tank sludge was used as the inoculation source, the nitrite-nitrogen concentration was 2.07 mg/L at 0.3V, whereas the nitrite-nitrogen concentration at other voltages was 1.1±0.15 mg/L. This significant difference suggests that the denitrification effect is poor at 0.3 V. For the aeration tank sludge inoculate, the optimum denitrification voltage was 0.5 V. For the root soil inoculate, the nitrite-nitrogen concentration at different voltages was 1.3±0.1 mg/L on average, and the optimum denitrification voltage was 0.5 V.

As it presented in Table 2, the removal rate of nitrate-nitrogen and the production of nitrite-nitrogen were significantly affected by different inoculates and voltages (*P* < 0.001), and there was a significant interaction between the two (*P* < 0.001).

**Table 2:** Effects of inoculation sources and voltages on nitrate nitrogen removal and nitrite nitrogen concentrations.

Independent Variable	Removal rate	Nitrite-nitrogen concentrations
Inculation source	*P* < 0.001	*NS*
Voltage	*P* < 0.001	*P* < 0.001
Inculation source * Voltage	*P* < 0.001	*P* < 0.001

Significance of treatments and interactions were determined by two-way ANOVA. *NS*: no significant difference

### 3.2. The total number of OTUs and the Venn diagram representation

**Fig 4:**
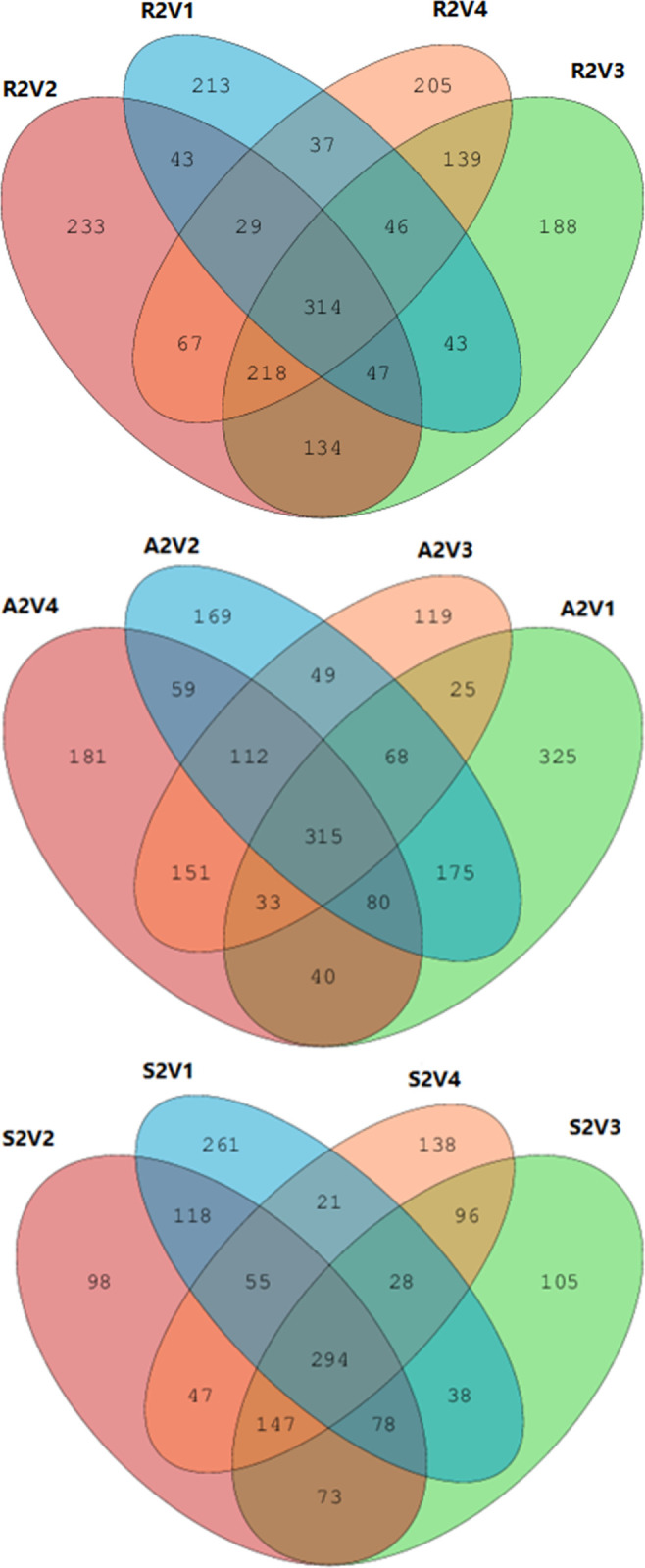
Venn diagram of OTUs for the three microbial inoculates under the four voltages for: (a) reflux sludge, (b) aeration tank sludge, and (c) root soil.

Using reflux sludge as the inoculate, the total number of OTUs across the four different voltages was 1,956, and the number of shared OTUs was 314, accounting for 16.0% (Fig 4A). At each respective voltage, the numbers of exclusive OTUs were 213, 233, 188, and 205, respectively, and the total number was 839, accounting for 42.9%. As demonstrated in Fig 4B, for the aeration tank sludge inoculate, the total number of OTUs across the four different voltages was 1,901, and the number of shared OTUs was 315, accounting for 16.6%. At each voltage, the numbers of exclusive OTUs were 325, 169, 119, and 181, respectively, or 794 in total, accounting for 41.8%. For the root soil inoculate, the total number of OTUs at the four different voltages was 1,597, with the shared number being 294, accounting for 18.4%. At each voltage, the numbers of exclusive OTUs were 261, 98, 105, 138, respectively, totaling 602 and accounting for 37.7%. From this, one can infer that a change in voltage has a significant effect on the structure of microbial communities in MEC devices initiated different inoculating sources.

### 3.3 Comparison of microbial commmunities among inoculates under optimum voltage

**Fig 5:**
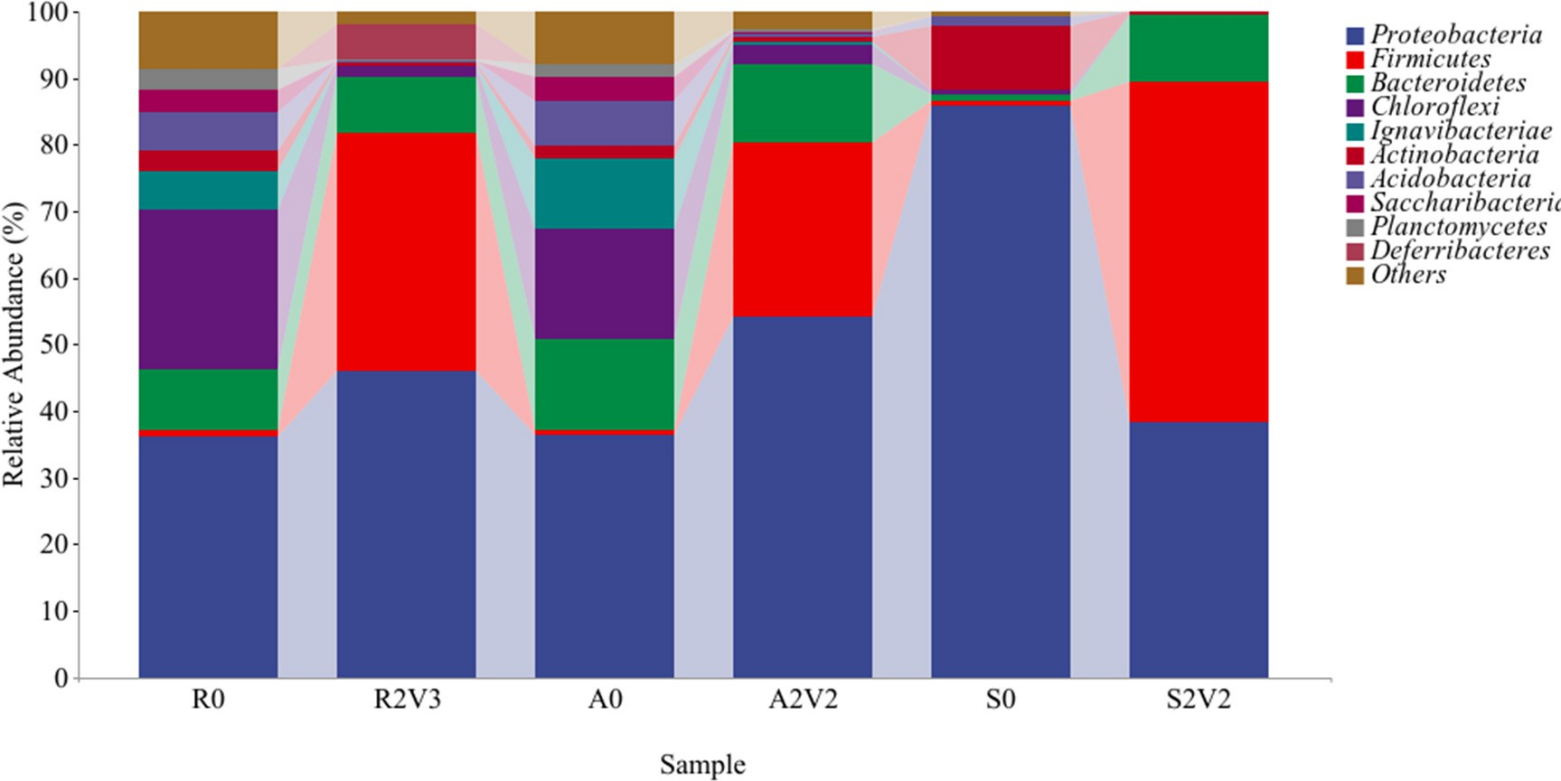
Microbial community in the simulated wastewater sample with different inoculates at the optimum voltage before and after culture at the phylum level (R0, A0 and S0 respectively presented inoculates of the returned sludge, aeration tank sludge and soil without applied voltage, while R2V3, A2V3 and S2V2 for the three inoculates at the optimum voltage).

We compared the microbial flora under the optimum voltage for the three different inoculates with the microbial flora in the inoculate at the phylum level. The bacterial community structures were dominated by the following phyla: *Proteobacteria*, *Firmicutes*, *Bacteroidetes*, *Chloroflexi*, *Ignavibacteria* and *Actinobacteria* (Fig 5). Differences in microbial community structure among the six samples indicate that the different conditions markedly influenced the relative abundance of various microorganisms. In the MEC denitrification reaction with reflux sludge as the inoculate, the proportion of *Proteobacteria increased* from 36.1% (R0) to 46.1% (R2V3) and *Firmicutes* from 1% (R0) to 35.6% (R2V3). in the MEC denitrification reaction initiated with aeration tank sludge, the proportion of *Proteobacteria increased* from 36.4% (A0) to 54.2% (A2V2) and *Firmicutes* from 0.7% (A0) to 26.1% (A2V2). For the root soil inoculate, the proportion of *Proteobacteria* decreased from 85.7% (S0) to 38.4% (S2V2) and *Firmicutes* increased from 0.8% (S0) to 50.9% (S2V2). Overall, *Proteobacteria* and *Firmicutes* were the main phyla of denitrifying bacteria achive in all MEC system.

### 3.4 Alpha diversity of the microbial community

**Fig 6:**
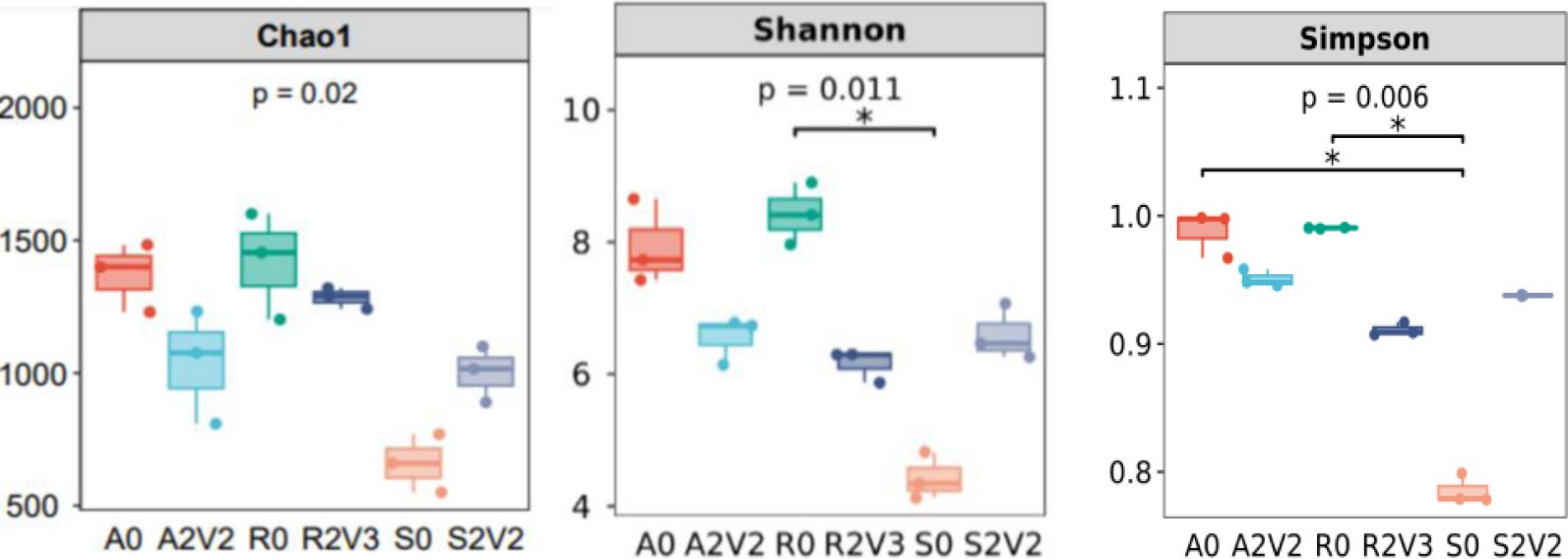
Alpha diversity indexes of different inoculates under the optimum voltage.

Six samples (R0, R2V3, A0, A2V2, S0 and S2V2) were examined in greater detail to characterize microbial community structure under different external voltages. The richness and diversity of the microbial communities evaluated using a series of indexes, i.e. the Chao 1, Shannon, and Simpson indexes, are visualized as box plots (Fig 6). The R0, R2V3, A0, A2V2, S0 and S2V2 samples exhibited Shannon indexes of 8.49, 6.12, 8.12, 6.51, 4.37 and 6.52, as well as Simpson indexes of 0.99, 0.91, 0.98, 0.95, 0.78 and 0.94, respectively. A low Shannon index is usually related to the low α-diversity of the microbial community [35]. Therefore, the lowest α-diversity was observed in S0 with the lowest Shannon and chao1. The bacterial diversity and richness of the samples in R0 and A0 were similar in terms at Shannon and Simpson indexes. Moreover, bacterial diversity and richness decreased when the voltage added (R2V3, A2V2). With the application of low current, the Chao1, Shannon and Simpson indexes for microorganisms with the soil inoculate, indicating that the low current can increase the diversity and richness of the microorganisms. Microbial communities for the reflux and aeration tank sludge inoculates showed different changes, As low current decreased microbial diversity to a certain extent. At the optimal nitrate-nitrogen removal effect using soil as the inoculate, we observed that the addition of the weak current (0.5V) promoted the accumulation of functional denitrifying bacteria, thus improving nitrate-nitrogen removal.

### 3.5 Beta diversity of the microbial community

**Fig 7:**
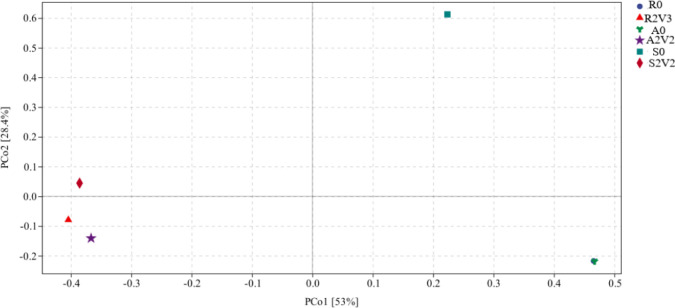
Beta diversity of different inoculates under the optimum voltage.

In the principal coordinate analysis (PCoA, Fig 7), different-colored points representes different samples, and point-to-point distance is proportional to differing species composition. PC1 and PC2 accounted for 28.4% and 53% of the varince. The applied voltage obviously affected community structure. The community from rhizosphere soil inoculate (S0) without applied voltage is far from those from the aeration tank and the returned sludge, indicating that its structural similarity is small. The distance between R0 and A0 (the returned and aeration tank sludge without applied voltage) samples is particularly small, indicating the structural similarity among the respective communities.

### 3.5 Heat map of microbial communities at the genus level

**Fig 8:**
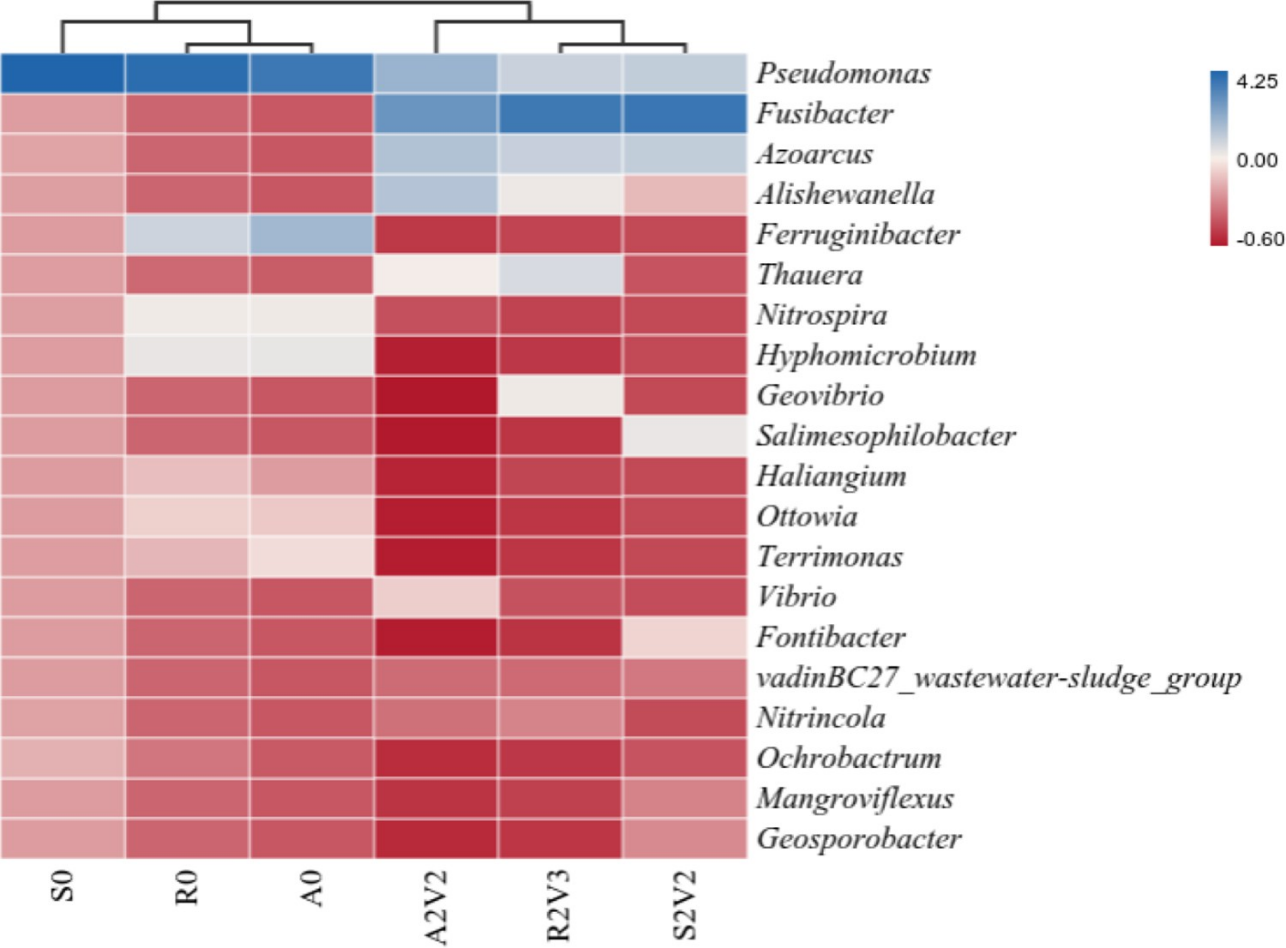
Heat map of microbial community structure at the genus level in the MEC.

To further analyze the differences in bacterial community composition of different microbial inoculates, a heat map (Fig 8) was constructed to cluster the top 20 bacterial genera. Different microbial inoculates and applied voltages caused different bacterial communities. *Pseudomonas*, *Fusibacter*, *Azoarcus*, *Alishewanella*, *Thauera* and *Ferruginibacter* had the highest relative abundance in S0, R0, A0, A2V2, R2V3 and S2V2. As shown in Fig 8, among the functional bacteria, *Pseudomonas* was the most common denitrifying bacterium [36]. *Fusibacter* is a common bacterium involved in hydrogen production [37]. *Pseudomonas* showed a trend of decline with voltage applied, in which the MEC using rhizosphere soil as inoculation source decreased most significantly from 77.0% to 12.6%. *Fusibacter* showed a significant increase with voltage, with inoculates from reflux sludge, aeration tank and rhizosphere soil increasing by 31.1%, 18.7% and 34.6%, respectively. The applied voltage also significantly increased the abundance of *Azoarcus* (a common Azotobacter) in MECs from the different inoculum sources. Therefore, microbial communities differed among the voltages applied, suggesting that characteristic hydrogenotrophic denitrifying populations were selected for and amplified to adapt to the operational conditions through cathode strengthening, leading to more efficient nitrate removal [38].

## 4. Conclusion

Nitrate removal performance varied with different microbial inoculates at different voltages in the denitrification reaction. A significant large difference in the diversity of microbial species at the phylum level was observed in the simulated wastewater sample with different inoculate sources at the optimum voltage before and after culture. The Chao1, Shannon and Simpson indexes showed that low current can increase the diversity and richness of the microorganisms in the soil-inoculated systems, while the reflux sludge and aeration tank sludge showed different changes. Low-current stimulation decreased microbial diversity to a certain extent. From the heat map of microbial community at the genus level, *Pseudomonas* showed a trend of decline with applied voltage, while *Fusibacter* showed a significant increase. The applied voltage significantly increased the abundance of *Azoarcus* in different inoculum sources. On the whole, the applied voltage increased the relative abundance of denitrifying bacteria.
